# In Vitro and In Vivo Germ Line Potential of Stem Cells Derived from Newborn Mouse Skin

**DOI:** 10.1371/journal.pone.0020339

**Published:** 2011-05-24

**Authors:** Paul W. Dyce, Jinghe Liu, Chandrakant Tayade, Gerald M. Kidder, Dean H. Betts, Julang Li

**Affiliations:** 1 Department of Animal and Poultry Science, University of Guelph, Guelph, Ontario, Canada; 2 Department of Physiology and Pharmacology, Schulich School of Medicine and Dentistry, The University of Western Ontario, and Children's Health Research Institute, London, Ontario, Canada; 3 Department of Anatomy and Cell Biology, Queen's University, Kingston, Ontario, Canada; University of Southern California, United States of America

## Abstract

We previously reported that fetal porcine skin-derived stem cells were capable of differentiation into oocyte-like cells (OLCs). Here we report that newborn mice skin-derived stem cells are also capable of differentiating into early OLCs. Using stem cells from mice that are transgenic for *Oct4 germline distal enhancer-GFP*, germ cells resulting from their differentiation are expected to be GFP^+^. After differentiation, some GFP^+^ OLCs reached 40–45 µM and expressed oocyte markers. Flow cytometric analysis revealed that ∼0.3% of the freshly isolated skin cells were GFP^+^. The GFP-positive cells increased to ∼7% after differentiation, suggesting that the GFP^+^ cells could be of *in vivo* origin, but are more likely induced upon being cultured *in vitro*. To study the *in vivo* germ cell potential of skin-derived cells, they were aggregated with newborn ovarian cells, and transplanted under the kidney capsule of ovariectomized mice. GFP^+^ oocytes were identified within a subpopulation of follicles in the resulting growth. Our finding that early oocytes can be differentiated from mice skin-derived cells in defined medium may offer a new *in vitro* model to study germ cell formation and oogenesis.

## Introduction

Skin-derived multipotent stem cells that are capable of differentiating into neural cells and adipocytes have been reported in mice [1] and pigs [2], [3]. These skin-derived stem cells have also recently been shown to form insulin producing cells [4]. In addition, stem cells with adipogenic, chondrogenic, and osteogenic differentiation potentials have been demonstrated in human skin [5]. These findings indicate that multiple classes of stem cells with differing differentiation potentials are present in the skin, making skin an affluent source of stem cells for the study of development and differentiation. In mice, these skin derived stem cells have been further characterized, and were shown to be of neural-crest origin [6].

We have previously reported on the isolation of stem cells from fetal porcine skin. These stem cells proliferate as non-adherent spheres. A subpopulation of these cells possess multi-lineage potential, as clonal populations expanded from individual cells were able to form neuron-, astrocyte-, and adipocyte-like cells upon induced differentiation [2]. More recently, we also found that a subpopulation of these skin-derived cells are capable of germ cell specification and can differentiate into oocyte-like cells (OLCs) when cultured in medium containing follicular fluid and fetal bovine serum [7]. However, it is not known if skin derived stem cells from other species also possess this differentiation potential, and whether this small subgroup of cells with germ cell potential is of *in vivo* origin or resulted from spontaneous *in vitro* reprogrammed skin cells remains unclear.

OCT4 is a member of the POU-domain family of transcription factors, which plays a crucial role in the maintenance of embryonic stem cell pluripotency and the establishment of the mammalian germ cell lineage (reviewed in [8]). Consistent with its roles, OCT4 is expressed in the early embryo, the expression is then down-regulated during gastrulation, and is thereafter confined to the germ cell lineage during later development [9], [10]. The distal element (DE, germline enhancer) of the *Oct4* regulatory region directs its expression in preimplantation embryos and germ cells, while the proximal element drives its epiblast-specific expression [10]. Thus, germline enhancer DE-driven *Oct4* expression offers an approach for monitoring germ cell formation. In this investigation, a transgenic mouse line bearing an *Oct4DE-GFP* construct (referred to as *Oct4-GFP*) was utilized to attempt to address two questions: do mouse skin-derived stem cells have germ cell potential similar to porcine skin-derived stem cells, and is this potential, if it exists, intrinsic to skin stem cells or does it arise during *in vitro* culture?

## Materials and Methods

### Stem Cell Isolation and Culture

All animal material-related experiments in the study were conducted according to Guide to the Care and Use of Experimental Animals of the Canadian Council on Animal Care, and have been approved by the University of Guelph Animal Care and Use Committee (08R055). Newborn female transgenic mice [Jackson Lab; 004654; (CBA/CaJ×C57BL/6J)F2]carrying the *Oct4- GFP* transgene were euthanized within 24 hrs of birth and the dorsal skin removed. Skin stem cells were isolated using a protocol by Toma et al. with modifications [1]. Skin samples from 4–5 pups were grouped and placed in Hank's balanced salt solution (HBSS) and cut into ∼1 mm square pieces using dissecting scissors. The samples were then washed 3× using HBSS, and re-suspended in 1 ml of 0.05% trypsin for 40 min. at 37 degrees Celsius. Following trypsinization, 1 ml of 0.1% DNase was added to the sample and incubated 1 min. at room temperature. Then 9 ml of HBSS was immediately added and the cells pelleted at 500× G for 5 min. Samples were then washed 1× with HBSS and 2× with DMEM-F12 with antibiotics. Following the last wash, the samples were mechanically dissociated in 1 ml of DMEM-F12 by pipeting. The partially dissociated samples were then filtered using a 40 µm cell strainer (BD). This was done by adding 9 ml DMEM-F12 to the dissociated cells and running them through the filter. This was followed by 10–15 ml of DMEM-F12. The resulting filtrate was then pelleted by centrifuging for 5 min. at 500× G. Each pellet obtained from 4–5 pups was then re-suspended in 10 ml stem cell medium (DMEM-F12 with 1× B27, 20 ng/ml EGF, and 40 ng/ml bFGF) and plated on a 10 cm dish (Sarstedt). At ∼72 hours after plating, the skin-derived stem cells grew as suspended spheres, which discriminated them from the rest of the skin cells (attached) in culture. To passage floating cell spheres, medium containing spheres was centrifuged and the pellet was gently dissociated using a large bore pipette. The cells were re-seeded in fresh stem cell medium as above. Cells were passaged every 4–6 days.

### Newborn Mouse Ovarian cell Isolation

Newborn mice were euthanized within 24 hrs of birth. Incisions were then made along the ventral body wall and ovaries were removed and placed into HBSS at room temperature. Following isolation, the ovaries were cleaned of connective tissues,and any fat using a stereoscope. The clean ovaries were then moved into 400 µl of 0.05% trypsin in a 4-well dish. The ovaries were incubated at 37 degrees Celsius. Every 5 min. the ovaries were pipetted until a single cell suspension was generated. Following dissociation, 200 µl of 0.1% DNase was added to the well and it was incubated for 1 min. at room temperature. The cells were then moved to a 15 ml tube and washed with 9 ml HBSS. The resulting cells were then counted on a hemocytometer and were ready to use for the ovarian re-association.

### Stem Cell differentiation and Ovarian Re-association

The isolated stem cells at passage two were pelleted at 500× G and re-suspended in 500 µl of PBS. The cells were dissociated to single cells by using vigorous pipetting. The cells were then washed in 9 ml of PBS and counted on a hemocytometer. For the stem cell only group (SC), and ovarian only groups, cells were plated at 0.6×10^6^ cells per well (500 µl) in differentiation medium which consists of TCM199 (no antibiotics) supplemented with 0.05 IU FSH, 0.03 IU LH, 3 mg/ml BSA, 5 µl/ml ITS, 0.23 mM sodium pyruvate, 1 mg/ml Fetuin, and 1 ng/ml EGF. For the co-culture, 0.3×10^6^ stem cells were mixed with 0.3×10^6^ ovarian cells in 500 µl differentiation medium and plated in a flat bottom suspension culture plate (Sarstedt). The cells were cultured at 37 degrees Celsius for 12 days, changing half the medium every 2 days. Spent medium was centrifuged and the pelleted cells returned to the culture dish. At the end of the 12 days of differentiation, the aggregates were trypsinized and large cells collected using a stereoscope and mouth pipette.

### RNA Isolation and RT-PCR

RNA was isolated using the RNeasy Mini Kit (Qiagen) according to the manufacture's protocol. RT-PCR on differentiated cultures was performed as previously described [2]. Briefly, RT-PCR on groups of 15 large cells or oocytes was performed by freezing cells in 7 µl of lysis buffer containing 14 U of porcine RNase inhibitor (Amersham) and 5 mM DTT (Invitrogen) at −80 degrees Celsius until use. Cells were then lysed by boiling for 1 minute and vortexing for 2 minutes (repeated for a total of 3×), and then stored on ice. Samples were then DNase treated by adding 1 µl 10× DNase buffer and 1 µl amplification grade DNase (Invitrogen) and incubating 15 min. at room temperature. 1 µl EDTA (25 mM) was then added and the samples were incubated for 10 min. at 65°C. RT was then performed by adding 0.5 µl H_2_O, 5 µl 5× buffer, 1.25 µl of random hexamer primers, 6.25 µl 2 mM dNTPs, and 1 µl MMLV reverse transcriptase to the sample. The samples were incubated at 25°C for 10 min., 37°C for 50 min., and 70°C for 15 min. Real-Time PCR was carried out on a Smart Cycler (Cepheid) by using the QuantiTect SYBR green PCR kit (Qiagen): 2.5 µl, for cell populations, and 3.1 µl for groups of 15 cells, of DNase treated cDNA (from a 25 µl RT reaction) was added to 12.5 µl of SYBR green mix and 0.3 µM each of forward and reverse primers (final volume 25 µl). Product sizes were confirmed on a 1% agarose gel. The identities of the products were confirmed by sequencing. Primers, expected product size, and accession numbers is presented in Table 1.

**Table 1:** RT-PCR Primers.

Target	Primers	Accession number	Product size
*Rp2*	5′-ggtggagaggagatggacaa-3′	Gi:6677794	246 bp
	5′-gcccaacacaaaacactcct-3′		
*Scp3*	5′-atgatggaaactcagcagcaagaga-3′	BC132079	125 bp
	5′-ttgacacaatcgtggagagaacaac-3′		
*Zp1*	5′-ggttgttttgtggtcctgct-3′	Gi:6677652	281 bp
	5′-tctagaacgtggcagccttt-3′		
*Zp2*	5′-aaggtcttgagcaggaacga-3′	Gi:76253946	252 bp
	5′-gggtggaaagtagtgcggta-3′		
*Zp3*	5′-gatccccgataagctcaaca-3′	Gi:6756082	283 bp
	5′-ggtttgagcagaagcagtcc-3′		
*Vasa*	5′-aggggatgaaagaactatggtc-3′	BC144760	175 bp
	5′-agcaacaagaactgggcact-3′		
*Gdf9b*	5′-attggagcgaaaatggtgag-3′	NM_009757	196 bp
	5′-aagtttccacatggcaggag-3′		
*Oct4*	5′-gaggagtcccaggacatgaa-3′	X52437	154 bp
	5′-agatggtggtctggctgaac-3′		
*DazL*	5′-cctccaaccatgatgaatcc-3′	Gi:31542548	228 bp
	5′-gggcaaaatatcagctcctg-3′		
*Fragilis*	5′-aacatgcccagagaggtgtc-3′	AY082484	174 bp
	5′-cttagcagtggaggcgtagg-3′		
*Dmc1*	5′-gggatacaaatgacaacaag-3′	D64107,CV876801	139 bp
	5′-cgaaattctccaaaagcttc-3′		
*Rec8*	5′-attcgacaccttttagaggctg-3′	NM_020002	203 bp
	5′-aagtctcctcgactgatctctg-3′		
*Stra8*	5′-acaacctaaggaaggcagtttac-3′	NM_009292	173 bp
	5′-gacctcctctaagctgttggg-3′		
*FigLa*	5′-acagagcaggaagcccagta-3′	NM_012013	205 bp
	5′-actcgcacagctggtaggtt-3′		
*Stella*	5′-tgagtttgaacgggacagtg-3′	Gi:20086232	200 bp
	5′-gatttcccagcaccagaaaa-3′		
*Nobox*	5′-acaaacgccatgagatttcc-3′	NM_130869	215 bp
	5′-aacagggccaggttctaggt-3′		
*Nanog*	5′-gcagaagtacctcagcctcc-3′	Gi:153791181	127 bp
	5′-ccactggtttttctgccac-3′		
*Sox2*	5′-ccaagatgcacaactcggag-3′	Gi:30024607	127 bp
	5′-ggtgctccttcatgtgcag-3′		
*GFP*	5′-cctgaagttcatctgcacca-3′		196 bp
	5′- ggtcttgtagttgccgtcgt-3′		

### Immunofluorescence

Cells were washed twice with PBS and fixed in 4% paraformaldehyde in PBS for 20 minutes. Cells were then washed three times in PBS with 0.1% Tween 20 and incubated for 10 minutes, and then for 20 minutes in PBS with 1% Triton-X-100. Next, cells were blocked for 2 hours in PBS with 5% BSA, and 0.05% Triton-X-100 (blocking solution), followed by an incubation with primary antibody, either 1∶500 anti-STELLA (Chemicon), 1∶400 anti-DAZL (Abcam), 1/300 anti-OCT4 (Cell Sciences), 1∶250 anti-GFP (Abcam) or 1∶400 anti-VASA (Abcam) for 2 hours at 37 degrees Celsius, or overnight at 4 degrees Celsius. Cells were then washed in blocking solution (PBS with 5% BSA and 0.05% Triton X 100), and incubated with 1∶500 phycoerythrin (PE)-conjugated goat anti-rabbit IgG or 1∶500 FITC-conjugated rat anti-mouse IgG for 1 hour at room temperature. This was followed with a blocking solution wash and incubation with 4′-6-diamidino-2-phenylindole (DAPI) for 1 minute, followed by washing three times with PBS-B. Cells were mounted using fluorescent mount medium (DakoCytomation) and viewed using an Olympus BX-UCB microscope and MetaMorph analysis software (Universal Imaging Corporation).

### Flow Cytometry

To analyze GFP in stem cell and differentiated samples the cells were mechanically dissociated to single cells and re-suspended in PBS. Gates were set based on non-transgenic stem cells (no GFP). Sorting was done in the same manner with cells collected instead of being disposed. SSEA1 (R&D Systems) was analyzed by mechanically dissociating the cells and blocking with PBS with 2% BSA and 1% FBS for 1 hour. The SSEA1 antibody was then added at a 1∶5 dilution and the samples were incubated for 1 hour at 4 degrees Celsius. The cells were then washed, by centrifugation, 3 times in the block buffer used above and then resuspended in 200–400 µl PBS for analysis. Analysis was performed using a FACScalibur flow cytometer (Becton–Dickinson) equipped with an argon laser emission wavelength of 488 nm. GFP and PE were identified using 530 nm and 585 nm band pass filters, respectively. Quantification analysis was completed using CellQuest software (Becton–Dickinson). Ten to twenty thousand events were acquired per sample with fluorescence measured on logarithmic scales. Forward and side light scatter gates were set to exclude dead cells, debris, and clumps of cells. In order to eliminate non-specific and Fc receptor-mediated binding of the antibodies, samples were blocked in serum before and during incubation with antibodies. Linear gates were set at 0.1%, on samples stained only with secondary antibody, or CD1 parallel cultures for GFP, and events corresponding to a fluorescence signal exceeding this percentage were measured as positive events. Autofluorescence was removed from the samples by setting gates on unstained controls.

### Immunohistochemistry of tissue sections

Paraffin embedded tissue sections were cut at 7 µm thickness and deparaffinized by treating with xylene and graded alcohols as per standard protocol. Sections were then washed for three minutes in PBS. Antigen retrieval was performed by immersing the slides in 85–90°C 10 mM citrate buffer with 0.05% Tween-20 (pH 6) for 12 min. The immersed slides were removed from the heating block and allowed to cool in the buffer for an additional 20 min. Slides were washed twice for 3 min. in PBS. Endogenous peroxidase was then blocked by incubating the slides in 3% hydrogen peroxide for 10 min. at room temperature. Slides were then washed twice in PBS for 5 min. and blocked in 5% BSA in PBS containing 0.025% Triton X-100 for 1 hour at room temperature. Following blocking, the slides were placed in a humidity chamber with primary antibody (1∶500 anti-GFP or 1° omitted in negative control) overnight at 4°C. Slides were rinsed three times for 10 min. with blocking buffer and incubated for 2 hr at room temperature with biotin conjugated secondary antibody (anti-goat 1/500). Slides were rinsed three times for 10 min. with PBS followed by incubation with conjugated horseradish peroxidase streptavidin for 20 min. in a humidity chamber. Slides were then rinsed briefly in PBS and reacted with DAB for 20 min. Slides were then washed for 5 min. in PBS and immersed in Harris hematoxylin for 5 min. followed by 2 min. in water, 4–5 dips in acid alcohol, brief rinse in water, and bluing in bluing agent for 2 min. The slides were then rinsed for 8 min. in running water. The slides were air dried and then mounted with fluorescent mount medium (Dako) and analyzed on an inverted microscope.

### Transplantation of stem cell and ovarian cell aggregates in vivo

The University of Guelph Animal Care Committee approved all transplantation procedures (AUP 08R015). Stem cells (0.3×10^6^ *Oct4-GFP*) were mixed with 0.3×10^6^ ovarian cells and plated in 500 µl of differentiation medium. The cells were cultured for 48 hrs prior to transplantation under the kidney capsule of *Rag2^−/−^gamma(c)^−/−^* immunodeficient mice (Taconic 004111). Immediately prior to transplantation, cells were pelleted in sterile PBS and 20 µl of the PBS was left on the cell pellet. Mice were anesthetized using Avertin (dosage 0.75 mg/g) and the ovaries were removed. The kidney was exposed, and a tear was made in the kidney capsule. The cell pellet was carefully inserted under the kidney capsule. Animals were euthanized 5–16 weeks post-transplantation and kidneys were harvested for analysis.

### Statistical Analysis

Experiments were repeated at least three times and the data were analyzed using a t-test (on comparison of germ cell marker expression levels between OLC and the oocyte groups) or ANOVA, followed by the Tukey test. Results were considered significant at P<0.05.

## Results

**Figure 1:**
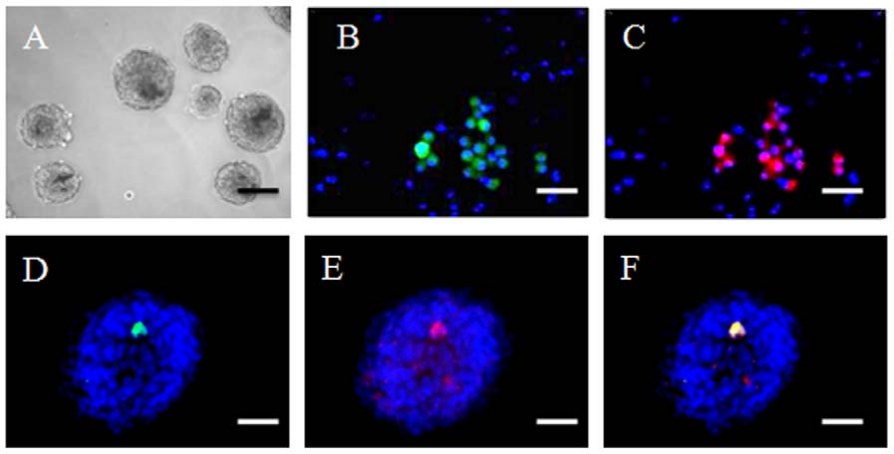
Morphology of skin-derived stem cell from newborn mice. A. Skin-derived spheres at P2 in culture. A subpopulation of the skin-derived stem cells is positive for GFP (direct fluorescence, B, D, F) and expresses OCT4 as revealed by immunofluorescence (red, C, E, F). B–F. Cells are strained with Hoechst to show nuclei. In F, the green GFP direct fluorescence and red GFP immunofluorescence are superimposed. Scale bars: A, 200 µm; B–F, 50 µm.

**Figure 2:**
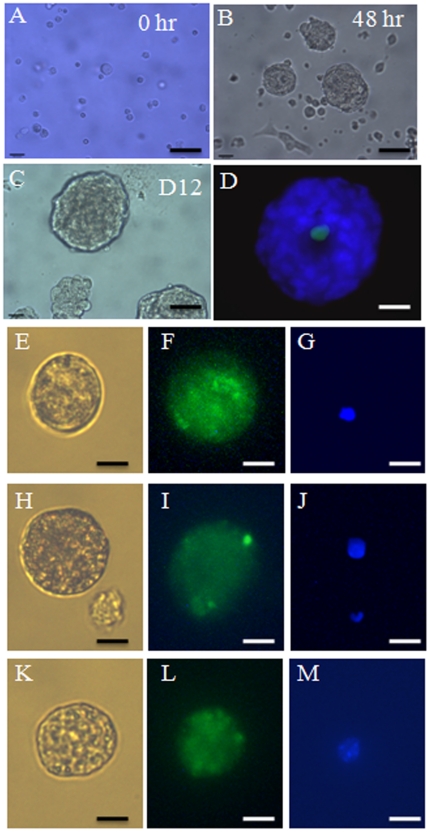
Morphology of skin-derived stem cells differentiating into germ cell-like cells *in vitro*. A. Dissociated newborn non-transgenic ovarian cells co-cultured with dissociated *Oct4-GFP* -expressing stem cells at 0 hr of induced differentiation. B. Ovarian and skin derived stem cell aggregates at 48 hrs of culture. C. The aggregates grew larger as the culture progressed with this image representing D12 of culture. D. A representative image of a GFP positive cell surrounded by GFP negative ovarian cells at D12 (cells are stained with Hoechst to show nuclei). E–M: Images of dissociated GFP-positive large cells and their nuclear staining (blue). Note that GFP is located in both nucleus and cytoplasm of the cells. Scale bars: A, 200 µm; B, 100 µm; C–D 50 µm; E–M, 25 µm.

**Figure 3:**
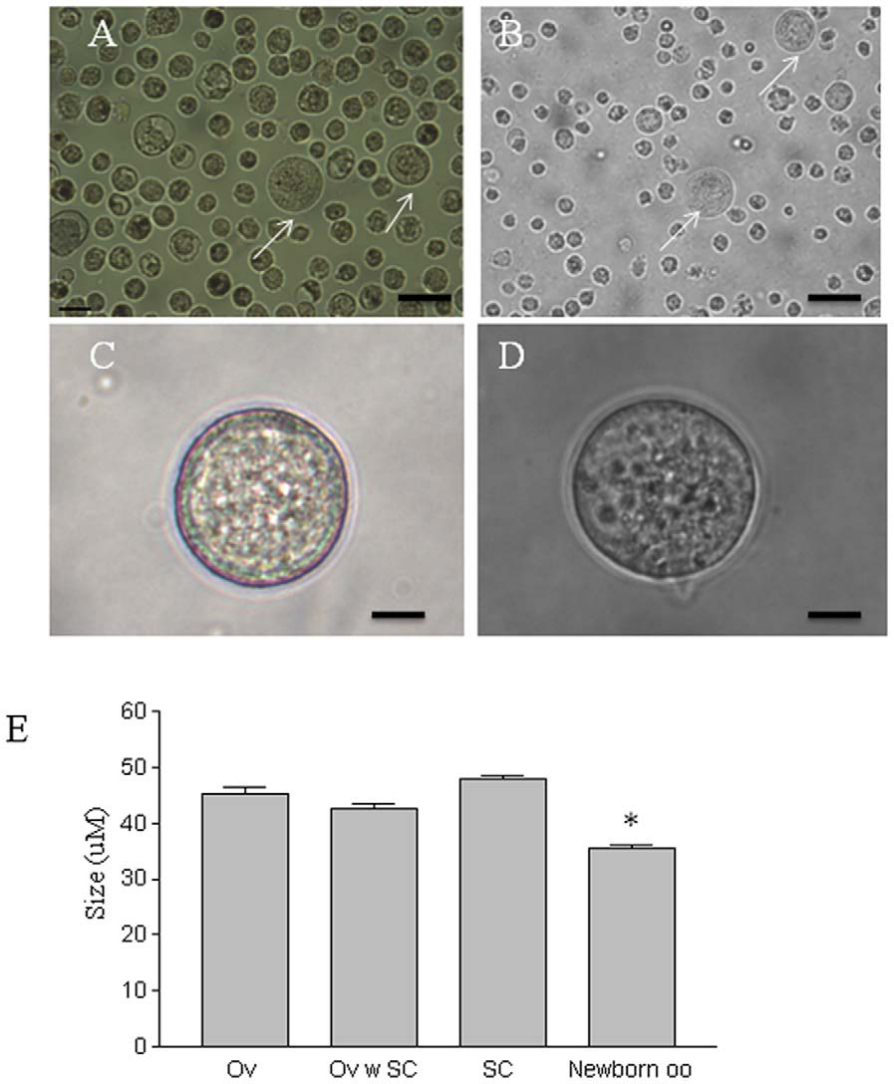
Oocyte-like cells generated from in vitro culturing. A. Oocyte-like cells (arrows) at D12 of differentiation following aggregate dissociation. B. Dissociated 12D cultured newborn ovarian cells as a positive control, arrows indicating oocytes. C. An oocyte-like cell generated from a skin-derived stem cell. D. A natural oocyte from a new born ovary (both C and D were cultured for 12 days). E. Mean diameters of oocytes from the newborn ovarian cell culture at D12 (Ov), D0 oocytes (newborn oo), oocyte-like cells from ovarian and stem cell co-culture (Ov w SC), and the stem cell group cultured for the same period of time (SC). 20–60 cells were measured per group. Data represent mean ± SE, * denotes significant difference from the Ov group. Scale bars: A,B 50 µm; C,D 15 µm.

**Figure 4:**
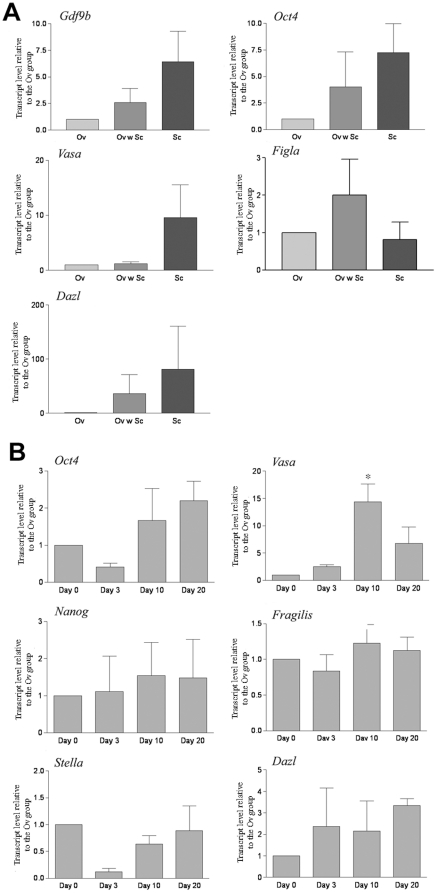
Expression of germ cell markers in germ cell like cells differentiated from skin derived stem cells. A. Comparison of germ cell marker expression levels in oocyte-like cells from ovarian and stem cell co-culture (Ov w SC) and the stem cell group (SC) to that of oocytes from newborn ovarian cell culture at D12 (Ov). Fifteen oocyte-like cells or oocytes were pooled and real-time PCR were performed after RNA isolation and reverse transcription. Transcript levels are expressed relative to the oocyte (Ov) group. Data represent mean ± SE of 3 experiments (data were analyzed using ANOVA). B. Germ cell marker expression in stem cell group during differentiation analyzed via RT-real time PCR. Data represent mean ± SE of 3 experiments. * indicates p<0.05.

In the mouse ovary, female germ cells proliferate and form cell clusters called germ cell cysts during the embryonic period [11]. Around the time of birth, pre-granulosa cells are involved in programmed cyst breakdown. This process is accompanied/followed by the formation of primordial follicles, although the mechanisms and factors governing folliculogenesis remain largely unknown [12], [13]. We first sought to utilize the newborn ovary as a microenvironment to induce germ cell/oocyte-like cell formation, reasoning that it may contain factors/interactions required for inducing oogenesis and follicle formation. Skin-derived stem cells were isolated from newborn mice transgenic for *Oct4-GFP* as previously described [2]. Figure 1A shows an image of the sphere stem cells. At passage two, a small subpopulation of these cells were positive for GFP (Figure 1B, D, F) and this co-localized with OCT4 staining (Figure 1C, E, F). Skin-derived stem cells were cultured with the dissociated ovarian cells from non-transgenic mice (Figure 2A). Forty-eight hours later, cell aggregates formed (Figure 2B), and larger aggregates were observed at Day 12 of culture (Figure 2C). A subpopulation of the aggregates was composed of a GFP positive cell surrounded by GFP-negative ovarian cells (Figure 2D). When dissociated, some of the cells were found to be ∼40 µM in diameter with a GFP positive subgroup (Figure 2E–M), confirming they were derived from the skin-derived stem cells. The same dynamic morphological changes (follicle-like structure formation, and oocyte-like cell formation) were evident in our positive control (Ov) in which newborn total ovarian cells were cultured alone (data not shown). To our surprise, similar to what was observed in the positive control group after cell dissociation (Figure 3B), at the end of the culture, a mixture of large and small cells was present in our negative control group (SC) in which skin-derived stem cells were cultured without ovarian cells (Figure 3A). The morphology of the oocyte-like large cells (Figure 3C) from the SC group was remarkably similar to the oocytes (Figure 3D) from the Ov group; both appeared to have a zona pellucida-like structure. Figure 3E shows the mean sizes of oocytes from newborn ovary cultures (Ov) and oocyte-like cells (OLCs) from the ovarian and stem cell aggregate culture (Ov w SC group; only those positive for GFP were counted) and SC group. The diameter of oocytes was increased from ∼35 µM (oocytes from newborn ovary, oo) to 45 µM after 12 days of culture in differentiation medium (Ov). No significant difference was observed in diameters among the Ov, Ov w SC, and SC groups. To study if OLCs express markers that are consistent with natural oocytes, 15 OLCs from each group were collected for RNA isolation. Figure 4A illustrates mRNA levels in OLCs from Ov w SC and SC groups in comparison to those of newborn ovary-derived oocytes cultured in the same conditions (Ov). While a large variation was observed, no significant difference was demonstrated among the three groups in terms of the expression level of oocyte markers *Oct4*, *Gdf9b*, *Vasa*, *Dazl* and *Figα*. The time line of the expression of these markers before and during induced differentiation was also investigated. Unexpectedly, the transcripts of all these markers were detected at day 0 of differentiation (Figure 4B).

**Figure 5:**
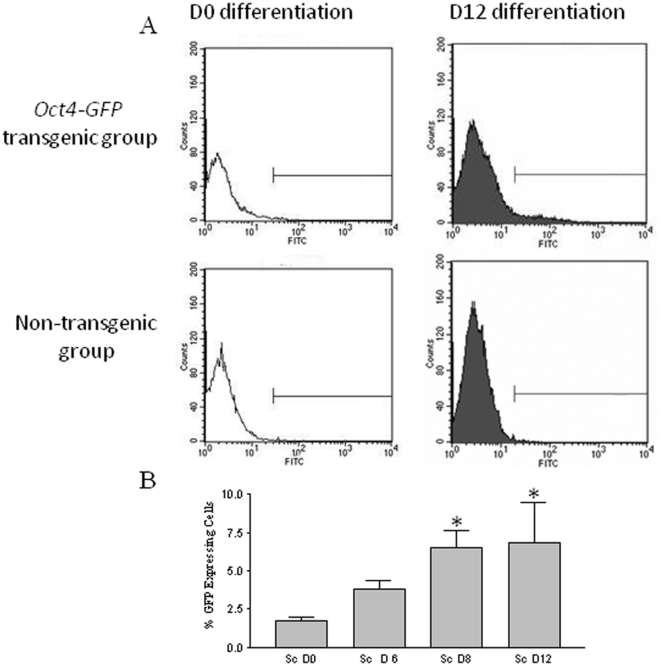
The percentage of GFP positive cells increases during induced differentiation as revealed by flow cytometry. A. Representative graphs of flow cytometric analysis of GFP expression in D0 and D12 cultured skin derived stem cells. Skin-derived cells from non-transgenic mice were used as negative controls. Horizontal and vertical axes indicate fluorescence and number of events, respectively. 10,000–20,000 events were analyzed per group/assay. Brackets indicate the expected fluorescence window for GFP. B. Percentage of GFP-positive cells (mean ± SE of three experiments). * denotes significant difference from D0.

**Figure 6:**
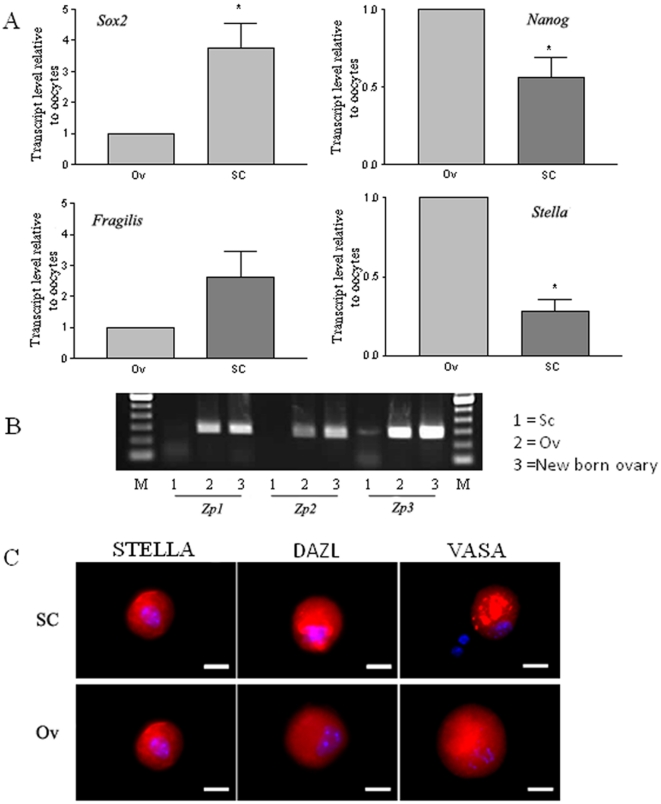
Comparison of germ cell marker expression in OLCs from the stem cell (SC) group with that of oocytes (Ov) after culture for 12 days. A. Real-time RT-PCR comparing the expression levels of *Sox2*, *Nanog*, *Fragilis*, and *Stella*. Data are mean ± SE of 3 experiments. (data were analysed using a t-test); * indicates P<0.05. B. RT-PCR showing the presence of *Zp3* mRNA but the absence of *Zp1* and *Zp2* mRNA in OLCs (SC group) after 12 days of differentiation. C. Expression (red) of DAZL, STELLA, and VASA in OLCs (SC group) using newborn ovarian oocytes (Ov group) cultured under the same conditions as the positive control. Blue shows the staining of the nucleus. Scale bars: C, 25 µm.

As similar outcomes were observed in the Ov w SC and SC groups, subsequent studies focused on OLCs generated from the SC group. To quantitatively monitor the induction of germ cell-like cells, flow cytometric analysis was performed to assess the percentage of GFP positive cells in our differentiation culture. As shown in Figure 5, the percentage of GFP positive cells increased gradually from day 0 to day 8 of differentiation, and was sustained for the rest of the differentiation period tested. The expression of additional germ cell markers was tested in OLCs generated from the SC group. As shown in Figure 6A, the expression of *Fragilis* is comparable between the two groups, the expression of *Nanog* and *Stella* is lower than that of oocytes, while the expression of *Sox2* is higher in OLCs from the SC group. RT-PCR also revealed that while *Zp1* and *Zp2* were not detectable, *Zp3* was expressed in the OLCs (Figure 6B). To study if OLCs express germ cell markers at the protein level, immunofluorescence analysis was also performed. Figure 6C confirms the expression of DAZL, VASA, and STELLA in OLCs derived from the SC group.

**Figure 7:**
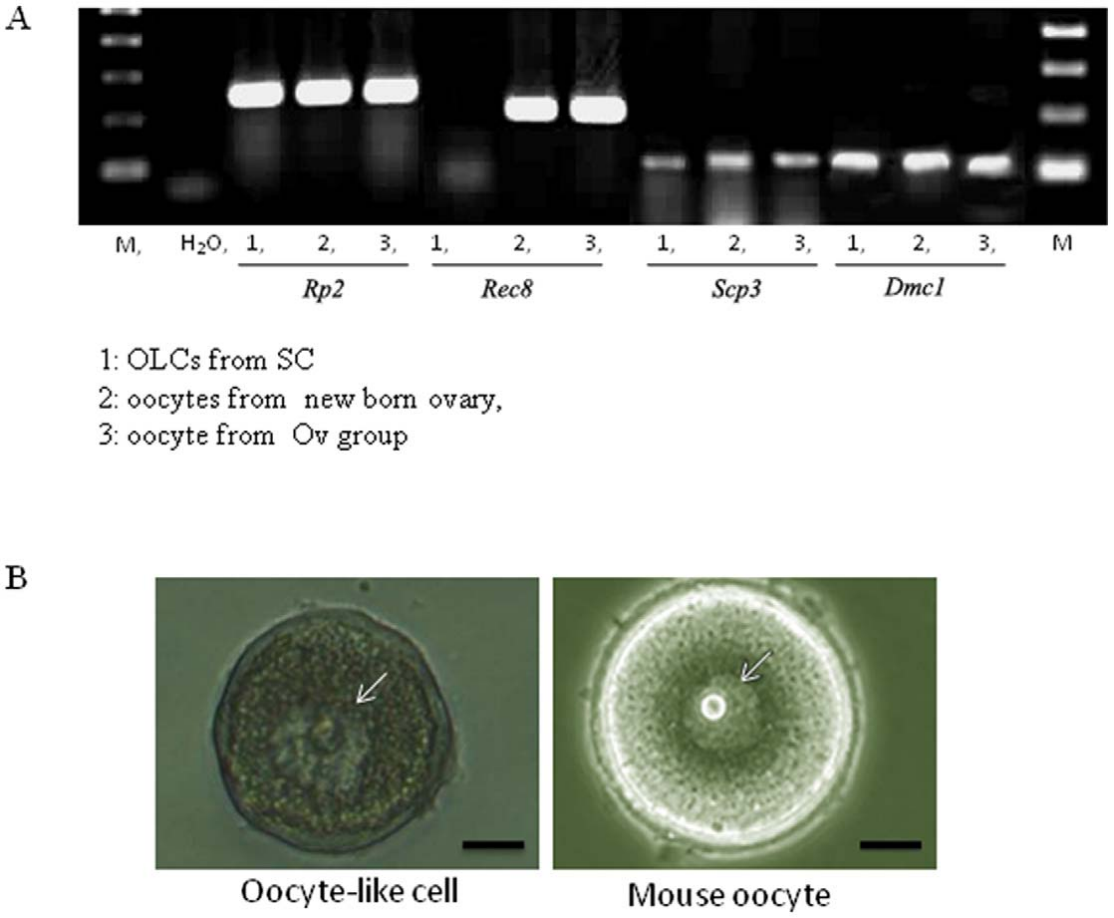
Detection of meiosis markers in OLCs by RT-PCR. A. Expression of *Dmc1*, *Scp3* and lack of expression of *Rec8* in the OLCs. *Rpii* was used as a loading control. B. Germinal vesicle-like structure (left, arrow) in an OLC similar to that of a natural oocyte (right).

Next we sought to study if these OLCs can undergo meiosis, a function which is unique to oocytes and male germ cells at later stages of their development. During meiosis REC8 is involved in chromosome reduction and segregation and co-localizes with SPC3 and DMC1 (Tarsounas, Morita et al. 1999). RT-PCR revealed that the transcript of *Rec8* was expressed in the Ov group, as well as the oocytes from the ovary of newborn mice but not in OLCs. *Scp3* and *Dmc1* transcripts are present in the OLCs and the two positive controls (Figure 7A). Immunocytochemistry of SCP3 was performed on chromosome spreads. It was found that the vast majority of SCP3-stained cells appeared to show discontinuous staining patterns that do not resemble any typical prophase stage (data not shown). Morphologically, some OLCs appeared to have a germinal vesicle (GV) like structure (Figure 7B). These data suggest some of the OLCs derived from SC can initiate meiosis, but they failed to progress beyond the MI stage in our current culture environment.

**Figure 8:**
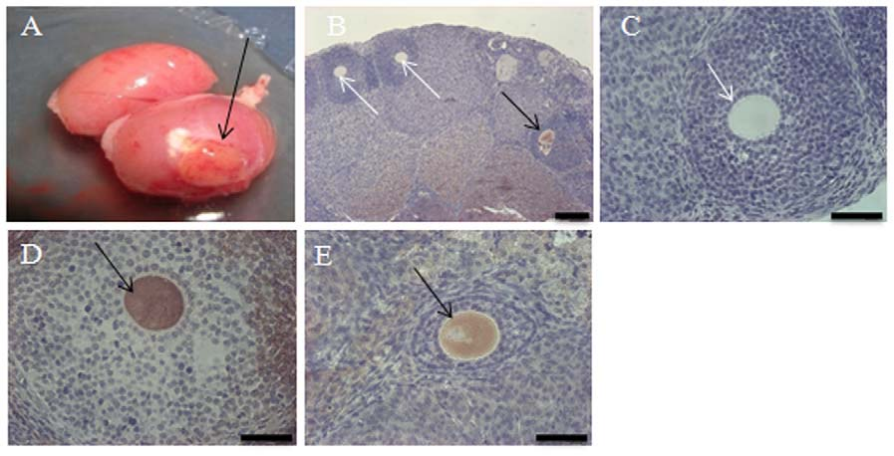
Formation of oocytes *in vivo* following transplantation of cell aggregates into immunodeficient mice. A. Growth under the kidney capsule (arrow) following 16 weeks of grafting. B. A follicle containing a GFP-positive oocyte (black arrow) and two follicles containing GFP-negative oocytes (white arrows). C. A follicle with a GFP-negative oocyte. D, E. Follicles containing positive oocytes (black arrows). Scale bars: B, 120 µm, C–E, 60 µm.

To study the *in vivo* germ cell potential of the newborn mouse skin-derived stem cells, we mixed 0.3×10^6^ stem cells with 0.6×10^6^ newborn ovarian cells and cultured 72 hours in the differentiation medium. Following culture, the reaggregated cells were transplanted under the kidney capsules of immunodeficient *Rag2^−/−^,gamma(c)^−/−^* ovariectomized mice. The kidney was chosen as the host organ of the transplant as it is one of the most heavily vascularized organs in the body and vascularization of the graft occurs within a short period of time so that the graft responds to the hormonal milieu of the host. Growths were identified under the transplanted kidney capsule after in vivo incubation (Figure 8A). Follicles with GFP-positive or negative oocytes, detected by immunostaining, were found within the growth (Figure 8B). Figure 8C is a follicle with GFP-negative oocyte, while Figures 8D and E show follicles with GFP-positive oocytes. The presence of a GFP-positive oocyte in the reconstituted transplanted ovarian structures provides evidence that under *in vivo* conditions, the newborn mouse skin-derived stem cells have the potential to form oocytes.

**Figure 9:**
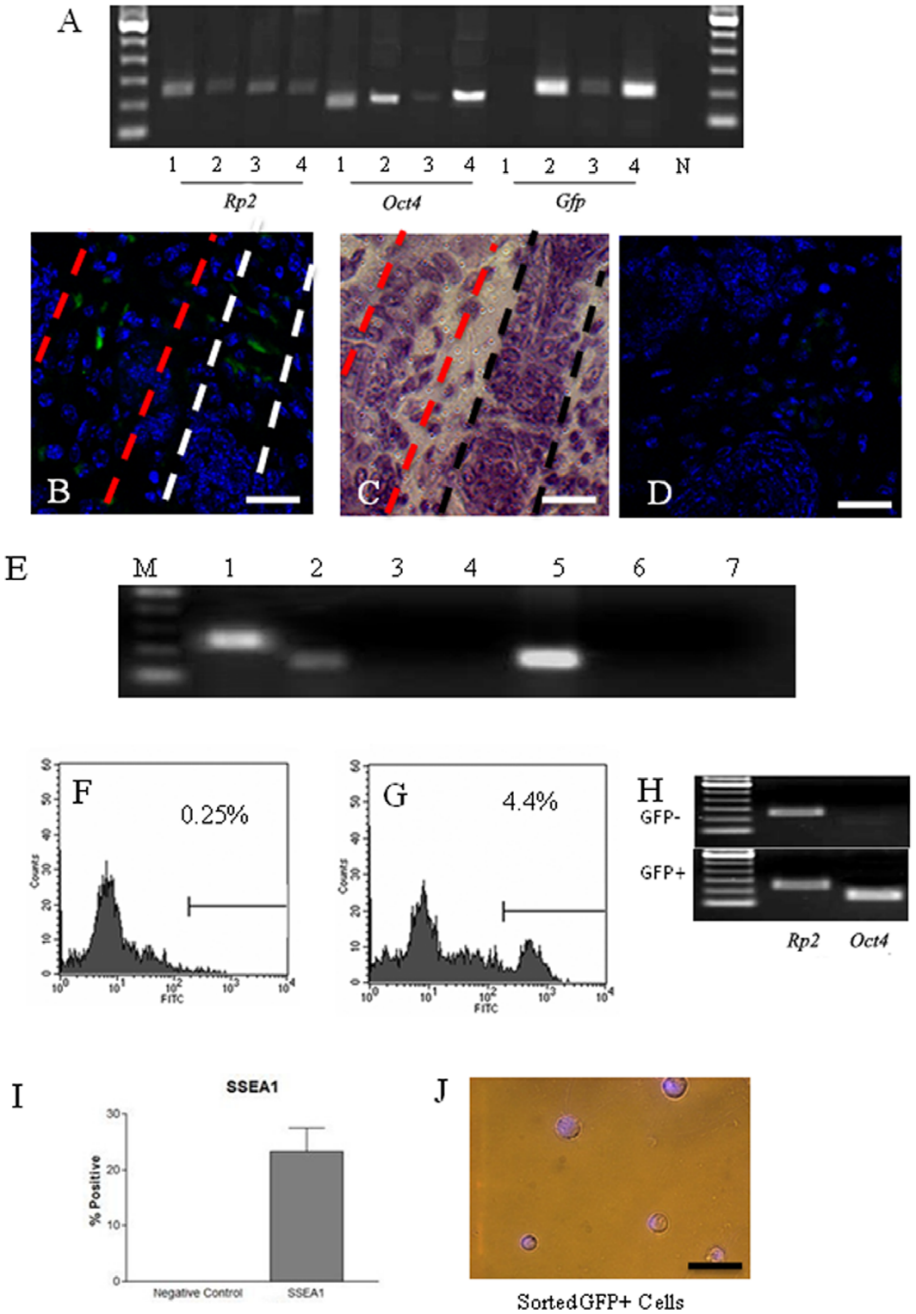
Expression of *Oct4-GFP* and other stem cell markers in skin-derived cells from *Oct4-GFP* transgenic mice. A. Detection of *GFP* and *Oct4* transcripts from newborn skin tissue. 1, ovary of non-transgenic mice (control); 2–4, skin of *Oct4-GFP* transgenic mice; N, negative control (PCR without reverse transcription for *Rp2*). B. GFP-positive cells located in a hair follicle of a new born mouse. C. H &E staining of the section in B showing the skin structure. D. Negative control in which the primary antibody was omitted. E. RT-PCR showing expression of a panel of genes in uncultured skin cells: 1, *Rp2*; 2, *Oct4*; 3, *Dazl*; 4, *Nanog*; 5, *Kit*; 6, *Stra8*; 7, *Scp3*. F,G. Representative flow cytometric analysis showing GFP-positive skin-derived stem cells at passage 0 (F) and 2 (G). Horizontal and vertical axes indicate fluorescence and number of events, respectively. H. RT-PCR detection of *Oct4* mRNA in the sorted GFP^−^ and GFP^+^ populations; *Rp2* was used as house keeping control. I. Detection of SSEA1 in skin-derived stem cells at passage 2 via flow cytometric analysis. Data are mean ± SE of 3 experiments. J. GFP positive stem cells (collected by cytometric flow sorting) depicting the morphology of germ cells including high nuclear to cytoplasm ratio. Scale bars: B–D and H, 50 µm.

We next examined whether the origin of *Oct4-GFP* positive cells is of an *in vivo* or *in vitro* nature. Immediately following isolation from the skin and before culture, less than 0.3% of cells analyzed by flow cytometry were positive for GFP. As it was difficult to clearly determine if they were background auto-fluorescing cells or real GFP-expressing cells at this low percentage, RT-PCR was performed on RNA isolated directly from newborn mouse skin. Figure 9A illustrates the detection of both *Oct4* and *GFP* transcripts in these skin samples, suggesting a small subgroup of cells from newborn mouse skin expresses *Oct4 in vivo*. This was confirmed by the localization of the GFP-positive cells in newborn mouse hair follicles (Figure 9B). Germ cell-specific markers such as *Dazl*, *Scp3*, *and Stra8* were not detectable in the uncultured skin cells (Figure 9E), suggesting that they are not committed to the germ cell path *in vivo*. Upon culture in the stem cell medium, GFP-positive cells increased from 0.25% to ∼4.4% at passage 2 (Figure 9F,G). RT-PCR confirmed that the GFP+ and GFP− cells are indeed *Oct4*+, and *Oct4*−, respectively (Figure 9H). We also cultured the GFP-negative cells for 7 days, and no GFP− positive cells were observed in the culture (data not shown), suggesting the increase in GFP+ cells in our whole population is due to the proliferation of preexisting GFP+ cells. A subpopulation of cells at passage 2 is also positive for SSEA1 (Figure 9I). The cells were then sorted to separate GFP-positive and negative groups. Figure 9J shows the morphology of the positive cells with large nuclear to cytoplasm ratio typical of oocytes.

## Discussion

Stem cell lines derived from the inner cell mass and epiblast of the mouse embryo, as well as germ cells, express *Oct4* [14]. Others and our group have previously detected *Oct4* positive cells derived from skin tissue [15], [16], [17], [18]. As endogenous *Oct4* expression can be driven by both distal element (DE, germline enhancer) and proximal element (epiblast regulatory element) [10] it was unclear if the OCT4-positive cells identified were at the state of stem cells from the inner cell mass, epiblast, or germ cells. Using the *Oct4DE-GFP* reporter transgenic mouse, the current study identified GF- expressing cells from cultured mouse skin-derived stem cells via flow cytometry. This finding suggests that the GFP positive cells are more closely related to cells of an early embryonic stage or germ lineage, rather than to cells from the epiblast stage. As expected, the GFP-positive cells express OCT4 at both mRNA and protein level, confirming the specificity of the reporter.

As pluripotent cells and early germ cells express similar groups of genetic markers, we focused our characterization on the large cells that morphologically resemble newborn ovarian-derived oocytes to test for *in vitro* germ cell potential. At the end of 12 days of differentiation, oocytes from the Ov group grew from 35.5 (D0) to 45.3 µm (D12) in diameter, suggesting that the medium supports *in vitro* growth of oocytes. On average, ∼10–50 OLCs can be obtained from the ∼0.6 million SCs (seeding density) in the SC group, whose size is comparable to oocytes from the Ov group. The size range of OLCs (∼35–60 µM in diameter) is in the same range as oocytes isolated from mouse ovaries 6–10 days post-partum [19]. In addition to the expression of *Oct4*, *Nanog*, *Fragilis*, *Sox2*, *Stella*, *Gdf9b*, *Vasa*, and *Dazl*, the OLCs also express *Figla* (factor in the germline, alpha). *Figla* encodes a germ cell specific basic helix-loop-helix transcription factor required for the expression of zona pellucida (ZP) genes [20], [21]. It is thought to also play a role in recruiting somatic cells to participate in follicle formation [21]. Disruption of *Figla* leads to a lack of ZP expression in the ovaries [21]. Consistently, *Zp3* transcript was detected in the OLC, and some of the OLCs show clear ZP morphology. However, *Zp1* and *Zp2* were not detectable in the OLCs, suggesting other factors governing the expression of these two members of the ZP family are still missing in the OLCs in our current culture system. The finding that some of the germ cell markers express at day 0 of differentiation was somewhat unexpected. However, previous studies have reported that many germ/pluripotent markers such as OCT4, VASA, FRAGILIS, DAZL, NANOG, and STELLA are expressed in both pluripotent stem cells and germ cells [22], [23], [24], [25]. It was suggested that this shared expression of genes is possibly due to both cells being primitive cells and therefore requiring the expression of similar groups of genes for maintenance of differentiation potency. This may explain our finding, as well as others [26], [27], [28] that most of the germ cell marker expression levels do not change significantly during stem cell to germ cell differentiation.

At the leptotene stage of prophase meiosis, each pair of sister chromatids forms a meiosis-specific longitudinal axial core to which the chromatin loops are attached. SCP3 is one of the elements of these axial cores [29], [30]. It is regarded as a marker for identifying meiotic transition as its expression is specific to meiosis and it is present from the initial step [31]. Later at the zygotene stage, the axial cores of each pair of homologous chromosomes become aligned in parallel, becoming the lateral elements of the synaptonemal complexes. DMC1, a mammalian homologue of RECA, is thought to play a role during chromosome synapsis and homologous recombination [32], [33], [34]. In addition, the meiosis-specific cohesin subunit REC8 is reported to be involved in sister chromatid separation in mice [35]. Our finding that OLCs express *Scp3* and *Dmc1*, together with the GV structure and the nuclear staining pattern of early meiosis suggests they are capable of initiating meiosis to the early stage. However these OLCs did not express *Rec8* and failed to enter the MII stage. These observations, together with the finding that most of the SCP3-stained cells do not exhibit typical prophase stage meiosis staining patterns, indicates that they did not progress to a later stage of meiosis, possibly due to lack of sufficient factors in the current culture system to support the further development of the OLCs. Interestingly, a previous study reported that *in vitro* produced ES-derived germ cells express SCP3 but do not correctly localized it, and furthermore, the chromosomes appeared more similar to somatic cell chromosomes than meiotic chromosomes [36]. Future studies involving the addition of selected factors to the currently rather simple medium will be important to help to identify those that are critical for proper meiosis. It has been suggested that only oocytes greater than 18 days old and larger than 60 µm are capable of going beyond the GV stage in mice [37]. The inability of our OLCs (mean diameter 48±0.7 µm) to complete meiosis is consistent with this notion.

The finding that the GFP positive cells increased in the SC group (meant to be a negative control in our preliminary study) was unexpected. It is generally thought that germ cell formation is a complex process governed by specific factors and carefully orchestrated interactions within a precise microenvironment. The medium used was a defined medium with EGF, LH, FSH, BSA, insulin, transferrin, selenium, and fetuin added into a M199 base. Although none of the main components has been previously reported to play a critical role in germ cell formation, it was reported that five germ cell transcripts (*Dkkl1*, *Hdc*, *Oct4*, *Zfp541* and *1700021K02Rik*) were altered by FSH in the testes of a hypogonadal mouse [38]. LH and FSH have also been suggested to be the prime regulators of primate spermatogenesis, as the combination of both hormones is important to achieve a quantitatively normal number of germ cells (reviewed in [39]. Comparison of global gene expression profiles revealed that insulin and the receptor for transferrin are both highly expressed in migrating PGCs [40], suggesting that they may play a role in early germ cell development. In addition, receptors for EGF, transferrin, and insulin were also found to be highly expressed in fetal mice ovaries [41], implying a possible role of the signaling pathway in oogenesis. It appears that removal of either hormones (LH, FSH) or EGF, although decreasing the number of GFP positive cells, was still able to support the retention of a certain percentage of GFP-positive cells (data not shown). It is possible that a small subpopulation of the skin-derived cells spontaneously step on to the germ cell path after they are removed from the *in vivo* skin tissue environment. The proliferation of these putative germ cells is then stimulated by these factors, either individually or in combinations. The significant increase of GFP positive cells in our experiment is consistent with this hypothesis.

Further culture of the aggregates with the current differentiation system failed to support the OLCs in growing larger (data not shown). Although expression of some granulosa cell markers such as *Fshr*, *Star*, and *Aromatase* was detected in the stem cell culture during differentiation (our unpublished data), the expression of *P450 C17*, which is also involved in the production of estradiol, was not detectable, nor was there estradiol in the spent medium. These, together with the lack of evidence for MII stage OLCs (including in the control Ov group) suggest that many more factors are required in the *in vitro* culture system to support functional follicle and oocyte formation.

Several hypotheses are offered to explain the germ cell potential of skin derived somatic stem cells. One possibility is that some of the PGCs are allocated to differentiating niches other than the gonads early during embryonic development and thereby end up residing in somatic tissues, where they retain germ cell potential. However, this is inconsistent with the fact that the estimated number of PGCs in mice ranges from approximately 100 to 5,000 during migration [42], while approximately 0.3% of the stem cells isolated were GFP positive. Another possibility is that a small population of PGCs “goes astray” and proliferates and these “lost” PGCs eventually populate the skin tissue. It does not, however, make physiological sense for the microenvironment within somatic tissues to sustain the survival and proliferation of PGCs when there are already an excess number of germ cells residing in the ovary. It is well known that less than one percent of oocytes within the mammalian ovary ovulate and fewer still will be fertilized to fulfill the task of transmitting genetic information to subsequent generations. A more favorable hypothesis is that there is a small subpopulation of uncommitted cells present in the skin, but these cells may never form germ cells in their native skin niche due to its suppressive environment for germ cell formation. However, when they are released from their restricted *in vivo* microenvironment, their germ cell potential could be unlocked during *in vitro* culture. Our finding that GFP positive cells increase during culture is consistent with this idea. Although ∼7% of the skin derived stem cells are GFP (thus OCT4) positive after *in vitro* culture, only 0.002–0.008% (10–50 out of 0.6 million) of the cells were obtained in the study. It appears that, although they are potentially pluripotent cells, only a small population of them are capable on becoming OLCs in the current culture condition, which highlights the need to optimize the medium in the future. In addition, it is known that primordial germ cells undergo a wave of apoptosis before they further differentiate into oocytes, thus it is possible that many of the germ cell-like cells did not make it to the OLC stage due to apoptosis. A recent hypothesis proposed that, during embryonic development, the epiblast acts as the source of all somatic stem cells following gastrulation. The epiblast, derived from the inner cell mass of the blastocyst, contains pluripotent epiblast stem cells (EpiSCs) that also retain germline potential [43]. It was suggested that some EPSCs may be deposited in peripheral tissues during gastrulation and that a very small population of these cells retain their pluripotent nature throughout development [43], [44]. The notion of uncommitted cells in skin, mentioned above, is in line with this hypothesis. It is possible that cells similar to these very small embryonic-like (VSEL) cells described by Ratajczak et al [44] are present in the skin of newborn mice, which, upon being placed in an appropriate environment, may first give rise to PGC-like cells and subsequently differentiate into OLCs. Are these uncommitted cells residing in the skin a physiologically relevant *in vivo* source of oocytes? Skin cells are less mobile than cells from other tissues such as bone marrow, closer to the external environment, and thus more prone to genetic mutations. If putative pluripotent cells are indeed present in somatic tissues, cells from skin may be the least likely source from which cells are recruited to contribute to the germline *in vivo*.
